# Anaphylaxis induced by mRNA COVID-19 vaccines: follow-up and booster dose after previous desensitization

**DOI:** 10.3389/falgy.2023.1056619

**Published:** 2023-05-03

**Authors:** Ibtihal AlOtaibi, Faisal Almuhizi, Shaonie Ton-Leclerc, Michael Fein, Christos Tsoukas, Lene Heise Garvey, Derek Lee, Moshe Ben-Shoshan, Ghislaine A. C. Isabwe, Ana M. Copaescu

**Affiliations:** ^1^ Division of Allergy and Clinical Immunology, Department of Medicine, McGill University Health Centre (MUHC), McGill University, Montreal, QC, Canada; ^2^ Division of Allergy and Clinical Immunology Department of Paediatrics, Central Second Health Cluster, Ministry of Health, Riyadh, Saudi Arabia; ^3^ Department of Internal Medicine, Security Forces Hospital Program, Riyadh, Saudi Arabia; ^4^ Faculty of Medicine, McGill University, Montreal, QC, Canada; ^5^ The Research Institute of the McGill University Health Centre, McGill University, McGill University Health Centre (MUHC), Montreal, QC, Canada; ^6^ Allergy Clinic, Copenhagen University Hospital, Gentofte, Denmark; ^7^ Department of Clinical Medicine, Copenhagen University, Copenhagen, Denmark; ^8^ Pharmacy Department, Montreal General Hospital, Montreal, QC, Canada; ^9^ Division of Allergy, Immunology and Dermatology, Montreal Children’s Hospital, McGill University Health Centre (MUHC), McGill University, Montreal, QC, Canada; ^10^ Centre for Antibiotic Allergy and Research, Department of Infectious Diseases, Austin Health, Heidelberg, VIC, Australia

**Keywords:** COVID-19, vaccine, mRNA, anaphylaxis, allergic reaction, desensitization, challenge, allergy

## Introduction

Although rare, anaphylaxis to the COVID-19 vaccine is a public concern. The rate of vaccine-related anaphylaxis in Canada is estimated to be 1.08 per 100,000 doses for the Pfizer-BioTech® vaccine and 0.77 per 100,000 doses for the Moderna® Spikevax COVID-19 vaccine (1). Recent data showed a variation in the incidence of vaccine-related anaphylaxis, depending on the definitions used for this acute reaction (2). Multiple mechanisms have been suggested to explain the underlying causes for the reported immediate reactions to the COVID-19 vaccines (3, 4). Studies that demonstrated tolerance to the second dose of the COVID-19 vaccine in patients with a history of anaphylaxis to the first dose (5–7) support a non-IgE-mediated mechanism. In November 2021, we shared our results of a successful desensitization protocol for the mRNA COVID-19 vaccine for six patients who had reported anaphylaxis to their first dose (8). With the evolving and reassuring data about the safety of subsequent doses in patients with a previous history of anaphylaxis, we re-evaluated the tolerance to the COVID-19 vaccine by performing a booster dose challenge.

## Methods

Patients were recruited as part of a large prospective 12-month COVID-19 vaccine study (ARCOV) (9). Individuals considered at risk for anaphylaxis to the COVID-19 vaccine were prospectively recruited. Six patients were selected based on a reported history of anaphylaxis to the first dose of the COVID-19 vaccine. The Brighton Collaboration case definition was used to define the levels of diagnostic certainty based on the reported symptoms (10). Brighton level 1 determines the highest level of diagnostic certainty that a reported case represents anaphylaxis; levels 2 and 3 are successively lower levels of diagnostic certainty; level 4 defines cases reported as anaphylaxis that do not meet the Brighton Collaboration case definition; and level 5 refers to cases that are neither reported as anaphylaxis nor met the case definition. Among our six patients who reported a history of anaphylaxis, four met level 2 Brighton's criteria, and two met level 3 and 4 criteria. As per our previously published protocol, PEG skin prick testing was performed during the initial assessment for all the patients with lower molecular weight (MW) PEGs: polyoxyl 35 hydrogenated castor oil (Cremophor EL) (527 mg/ml), PEG 300 (100% wt./vol), PEG 3,000 (50% wt./vol), PEG 3,350 (50% wt./vol), polysorbate 80 (20% wt./vol), and high MW PEG 20,000 (0.01%, 0.1%, 1%, and 10% wt./vol) (8). All six patients had safely received the second dose of the culprit COVID-19 vaccine using a desensitization protocol consisting of a graded dose administration followed by a 60-min observation period. Three patients received the Moderna® mRNA-1,273 and three the Pfizer-BioNTech® BNT162b2 vaccine. We offered a booster dose of the Pfizer-BioNTech® COVID-19 vaccine using a two-step blinded placebo-controlled challenge with a 1-hour observation period in a monitored setting. We defined tolerance to a subsequent dose as either (1) no immediate symptoms after the COVID-19 vaccine dose administration or (2) symptoms that were mild, self-limited, and resolved with oral antihistamines alone.

## Results

All six patients were females aged 33–66 years. Five patients had a past medical history of drug or vaccine allergy. PEG SPT was initially performed and resulted in delayed positive in 2 patients. The first patient had a delayed positive (3 h) to Cremophor EL and the second patient had a delayed positive (5 h) to Cremophor EL, PEG 300, PEG 3,000, and PEG 3,350. Skin testing was repeated for the second patient, resulting in an immediate positive for PEG 300.

Of the six patients administered the booster vaccine doses, one received a one-step challenge in the community and reported no adverse reactions, and one refused the 3rd vaccine dose (Table 1). The remaining four patients completed the two-step blinded placebo-controlled challenge in a controlled outpatient setting. One patient taking regular doses of daily prednisone 5 mg was premedicated with prednisone 10 mg for three days, rupatadine 20 mg and acetaminophen 975 mg on the day of the challenge. She completed the challenge without any severe systemic reaction. However, she developed hives on her left arm and right leg 20 min after completing the challenge. The urticarial skin eruption persisted, and she required prolongation of the prednisone 10 mg and rupatadine 20 mg for one more day. A second patient reported symptoms 20 min after receiving 0.1 ml of saline (placebo). She complained of itchy throat and ears, difficulty breathing, swelling inside her ears and feeling very uncomfortable. The patient was informed that she had been given a placebo in this context. After reassurance, she was able to complete the challenge safely. Twenty-five minutes after the last dose, she reported mild back itching and received 20 mg of cetirizine which resolved her symptoms. The remaining two patients completed the challenge uneventfully.

**Table 1:** Patients demographics, reaction to the first dose, desensitization and challenge outcomes.

Patient	Gender/age	Comorbidities	Signs and symptoms	Brighton's level^a^, NIAID/FAAN^b^, WAO^c^	Epinephrine received	PEG skin testing result	mRNA Vaccine type	Latency between first reaction and desensitization	Desensitization outcome	Booster dose	Latency between index reaction and booster dose^*^	Challenge outcome
1	F 49	Anaemia Neuropathic pain	15 min: Generalized erythema Urticaria Dyspnea	Level 2, Yes, Yes	No	Delayed positive (3 h) Cremophor EL	mRNA-1,273 (Moderna Spikevax)	5 months	Subjective pruritus without skin rash – treated with Cetirizine 20 mg PO. Cough and globus sensation – reassurance	BNT162b2 (Pfizer-BioNTec) 2- step Challenge	16 months	2- step Challenge in outpatient setting: Tolerated challenge without any symptoms.
2	F 66	Steroids-dependent spondylarthritis Colitis	30 min: Generalized urticaria and angioedema lasted for 2 days^d^	Level 4, No, No	No	Delayed positive (5 h) Cemophor EL PEG 300 PEG 3,000 PEG 3,350 Repeated: Positive (15 min) PEG 300	mRNA-1,273 (Moderna Spikevax)	3 months	Tolerated desensitization without immediate symptoms. Mild pruritis 3 days after desensitization.	BNT162b2 (Pfizer-BioNTec): 2- step Challenge in Clinic	14 months	2-step challenge in outpatient setting: - Premedicated with Prednisone 10 mg for 3 days, Rupatadine 20 mg and Tylenol 975^e^. - Had visible hives on arm and leg 20 min after last step of challenge (lesions lasted for 1 day).
3	F 64	CSU Diverticulosis Hypothyroidism Oral lichen planus	15 min: Urticaria, itchiness of her feet, hands, arms, and throat with dizziness	Level 2, Yes, Yes.	Yes	Negative Histamine – Negative	mRNA-1,273 (Moderna Spikevax)	3 months	Headache – treated with Acetaminophen Hives on the third and fourth day.	mRNA (Moderna)	9 months	1- step challenge in the community vaccine center: Self-resolved Throat tightness 15 min after.
4	F 35	None	15 min: Generalized itchiness with Throat itching and difficulty swallowing, nausea, vomiting and dizziness	Level 2, Yes, Yes	No	Negative	BNT162b2 (Pfizer-BioNTech)	4 months	Pruritus, dizziness and drop in systolic BP – treated with Cetirizine and IV fluid	BNT162b2 (Pfizer-BioNTec): 2- step Challenge	13 months	2- step Challenge in outpatient setting: Tolerated challenge without any symptom
5	F 57	Asthma Ehlers-Danlos Syndrome Osteopenia	30 min: Facial numbness and swelling, cough and difficulty breathing, hoarseness and chest pain	Level 1, Yes, Yes	No	Negative	BNT162b2 (Pfizer-BioNTech)	4 months	Tolerated desensitization without immediate symptoms. Generalized non-severe MPE after 1 week: lasted for 3 weeks with Skin desquamation.	Refused		
6	F 33	Asthma Depression Post-traumatic stress disorder	10 min: Throat itching and swelling, wheezing, vomiting, generalized numbness	Level 2, Yes, Yes	Yes (5 doses) 3 in vaccine center and 2 in ambulance	Negative	BNT162b2 (Pfizer-BioNTech)	4 months	Tolerated desensitization with mild neck itching.	BNT162b2 (Pfizer-BioNTec): 2- step Challenge in Clinic	13 months	2- step Challenge in the outpatient setting: Reacted 20 min after placebo: itchy throat and ear, evolving towards difficulty swallowing, feeling uncomfortable and dizzy. Reassured after knowing she received a placebo. Completed 2 steps challenge, 25 min after the last dose, had mild back itching, treated with an antihistamine.

CSU, chronic spontaneous urticaria; mRNA, messenger RNA; MPE, morbilliform drug eruption; NIAID/FAAN, national institute of allergy and infectious diseases and food allergy and anaphylaxis network.

^a^ The Brighton Collaboration case definition uses combinations of symptoms to define levels of diagnostic certainty. Brighton level 1 represents the highest level of diagnostic certainty that a reported case represents anaphylaxis; levels 2 and 3 are successively lower levels of diagnostic certainty; level 4 is a case reported as anaphylaxis, but that does not meet the Brighton Collaboration case definition, and level 5 is a case that was neither reported as anaphylaxis nor met the case definition.

^b^ YES: patient meets the National Institute of Allergy and Infectious Diseases (NIAID) and the Food Allergy and Anaphylaxis Network (FAAN) criteria for anaphylaxis.

^c^ YES: patient meets the World Allergy Organization (WAO) anaphylaxis definition criteria.

^d^ This patient premedicated herself before the first dose with oral diphenhydramine 25 mg and oral rupatadine 10 mg.

**^e^**The premedication was taken by the patient at home before the challenge and was not instructed by us.

*Latency is defined as the time, in months, between the first reaction and the moment of the challenge.

## Discussion

**Figure 1:**
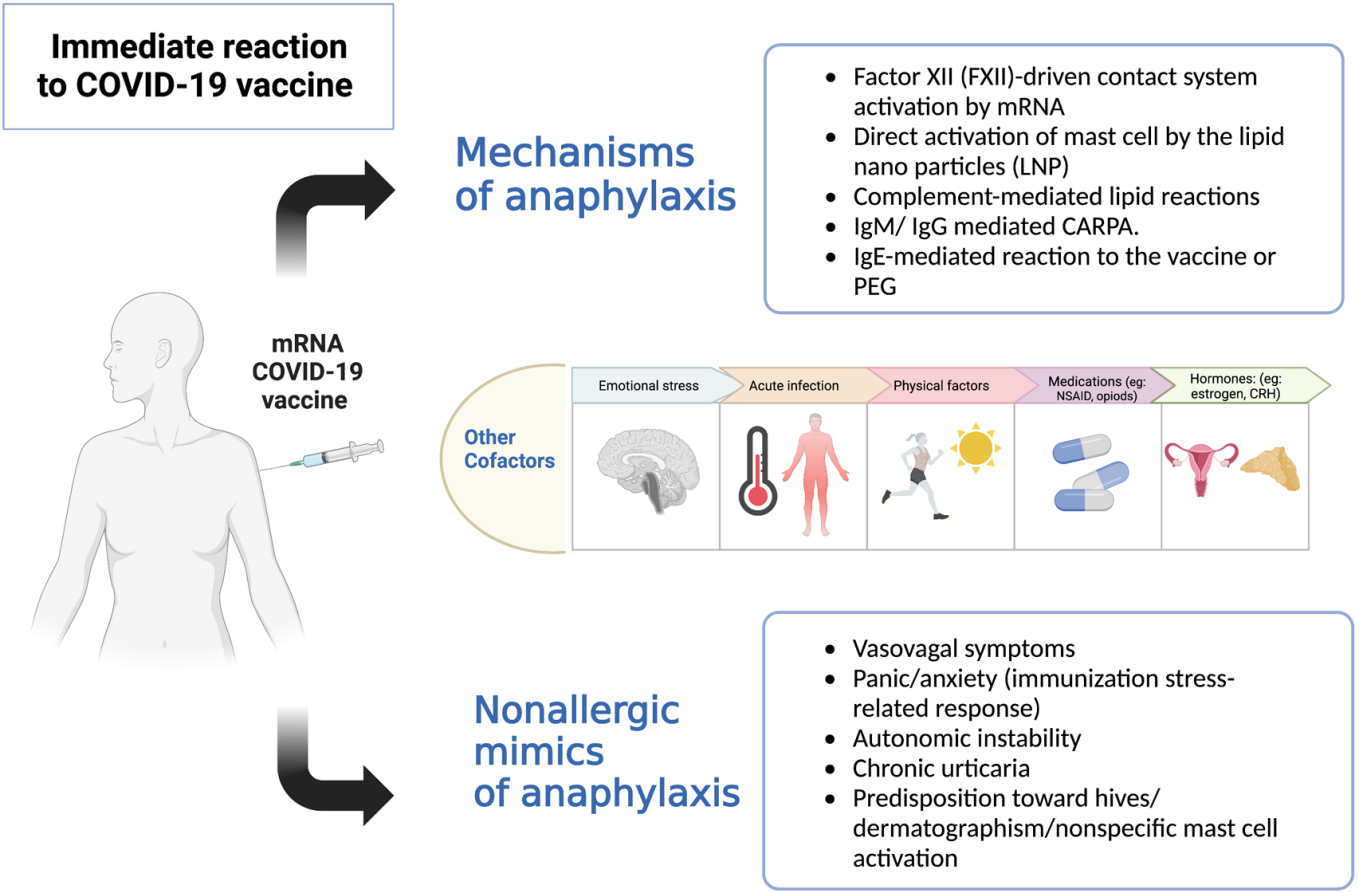
Potential mechanisms of immediate reactions to the COVID-19 vaccines (4). **Legend:** Multiple suggested mechanisms of anaphylaxis to the COVID-19 vaccine: (1) Exogenous nucleic acids activate factor XII leading to contact activation and production of bradykinin, causing increased vascular permeability, angioedema, hypotension and bronchoconstriction. (2) Direct activation of mast cells by lipid nanoparticles (LNP) via various receptors, e.g., opioids receptor, mast cell related G protein-coupled receptors X2 (MRGPRX2). (3) Lipid nanoparticles (LNP) in mRNA vaccine include neutral lipids, which may activate anaphylatoxins complement component 3a (C3a) and complement component 5a (C5a), which leads to the release of histamine, leukotrienes, prostaglandins that can lead to flushing, hives, hypoxia, vasodilatation, and hypotension. (4) Forming previous antibodies (IgM, IgG) against PEG or LNP can bind to complement and cross-link with the Fc receptor on mast cells leading to degranulation. (5) IgE against PEG on vaccine can cause anaphylaxis in patients with true PEG allergy. Host cofactors (genetic and environmental) can modify mast cell activation and increase predisposition to an immediate reaction. Other nonallergic reactions mimicking anaphylaxis should be considered in assessing patients with immediate reactions.

Rechallenging patients with a history of anaphylaxis to the mRNA CoV-2 vaccine is still discouraged because of the unknown safety of the procedure and the lack of understanding of the possible mechanisms involved (Figure 1) (4, 11). We previously demonstrated the safety of administrating the second vaccine dose using desensitization or a graded dose protocol. This cautious approach aimed at ensuring that patients could safely complete their vaccinations (8). In this study, we safely dispensed the vaccine booster in our small cohort by administering a 3rd vaccine dose in a 2-step challenge protocol. Our findings suggest that non-IgE-mediated mechanisms, including potentially direct mast cell activation, could explain the initial reactions (Figure 1) (4). Similar results were described by Krantz et al. when they challenged eight patients with a prior history of anaphylaxis (6). Their data revealed that serum tryptase levels at the reaction time were normal when collected. Unfortunately, we did not obtain serum tryptase in our patients.

Administering a new vaccine to patients with a previous history of anaphylaxis, including vaccines and drugs, is challenging as it requires prompt action to identify and treat possible symptoms of anaphylaxis. Interestingly, in our study, 2 out of 6 patients required epinephrine to manage their initial reaction, and one received five doses of epinephrine before arrival at the hospital. This patient had a history of anxiety and post-traumatic stress disorder (PTSD) and reacted to the placebo during the challenge. This case demonstrates the often-encountered dilemma of distinguishing patient anxiety and psychosomatic symptoms mimicking “allergic reactions” from the true anaphylaxis (12).

The European Academy of Allergy & Clinical Immunology has recommended skin testing with PEG for patients with an allergic reaction to the COVID-19 vaccine (13). However, several studies found that patients with positive skin tests tolerated the vaccination and some patients with negative skin tests developed a reaction (14). The accuracy of PEG skin testing in the context of a reported mRNA vaccine reaction is yet to be established (3). PEG-allergic patients can tolerate the mRNA vaccine (15). However, this tolerance of mRNA vaccines does not rule out PEG allergy, and patients who tolerate the mRNA vaccines may nevertheless experience severe reactions to PEG (3). Performing this testing on our patients did not assist us in determining the tolerance of the second dose of the RNA COVID-19 vaccination (14, 16). In our view, a delayed positive skin test is not a sign of PEG hypersensitivity, and the utility and validity of testing remain unknown.

We revisited the initial reactions and used different diagnostic criteria for anaphylaxis (Table 1). All patients met Brighton's criteria with different diagnostic certainty. However, one patient did not meet the NIAID or WAO Criteria (2020) (16, 17). The anaphylaxis definition varies depending on the diagnostic criteria used. Furthermore, Brighton's criteria overestimate the anaphylaxis prevalence (2). We believe that genuine anaphylactic reactions to the COVID-19 vaccination are infrequent. A case-by-case evaluation should be performed to confirm or refute the initial anaphylactic diagnosis and thus offer the opportunity for a vaccine challenge allowing the completion of the scheduled immunization program.

## Conclusion

Patients with a history of possible anaphylaxis should be assessed in an allergy unit to validate the initial reaction. A history of suspected anaphylaxis to the COVID-19 vaccine may not be a contraindication for receiving subsequent vaccine doses in an allergist-supervised setting. Large-scale studies are required to understand better the underlying mechanisms for the immediate reactions reported to the COVID-19 vaccine.

## Data Availability

The authors confirm that the results supporting the findings of this study are available within the article and/or its supplementary materials. Other data supporting the findings of this study are available from the corresponding author on request.
